# Vector competence of biting midges and mosquitoes for Shuni virus

**DOI:** 10.1371/journal.pntd.0006993

**Published:** 2018-12-07

**Authors:** Tim W. R. Möhlmann, Judith Oymans, Paul J. Wichgers Schreur, Constantianus J. M. Koenraadt, Jeroen Kortekaas, Chantal B. F. Vogels

**Affiliations:** 1 Laboratory of Entomology, Wageningen University & Research, Wageningen, The Netherlands; 2 Department of Virology, Wageningen Bioveterinary Research, Wageningen University & Research, Lelystad, The Netherlands; 3 Laboratory of Virology, Wageningen University & Research, Wageningen, The Netherlands; University of Veterinary Medicine, Vienna, AUSTRIA

## Abstract

**Background:**

Shuni virus (SHUV) is an orthobunyavirus that belongs to the Simbu serogroup. SHUV was isolated from diverse species of domesticated animals and wildlife, and is associated with neurological disease, abortions, and congenital malformations. Recently, SHUV caused outbreaks among ruminants in Israel, representing the first incursions outside the African continent. The isolation of SHUV from a febrile child in Nigeria and seroprevalence among veterinarians in South Africa suggests that the virus may have zoonotic potential as well. The high pathogenicity, extremely broad tropism, potential transmission via both biting midges and mosquitoes, and zoonotic features warrants prioritization of SHUV for further research. Additional knowledge is essential to accurately determine the risk for animal and human health, and to assess the risk of future epizootics and epidemics. To gain first insights into the potential involvement of arthropod vectors in SHUV transmission, we have investigated the ability of SHUV to infect and disseminate in laboratory-reared biting midges and mosquitoes.

**Methodology/Principal findings:**

*Culicoides nubeculosus*, *C*. *sonorensis*, *Culex pipiens pipiens*, and *Aedes aegypti* were orally exposed to SHUV by providing an infectious blood meal. Biting midges showed high infection rates of approximately 40–60%, whereas infection rates of mosquitoes were lower than 2%. SHUV successfully disseminated in both species of biting midges, but no evidence of transmission in orally exposed mosquitoes was found.

**Conclusions/Significance:**

The results of this study show that different species of *Culicoides* biting midges are susceptible to infection and dissemination of SHUV, whereas the two mosquito species tested were found not to be susceptible.

## Author summary

Arthropod-borne (arbo)viruses are notorious for causing unpredictable and large-scale epidemics and epizootics. Apart from viruses such as West Nile virus and Rift Valley fever virus that are well known to have a significant impact on human and animal health, many arboviruses remain neglected. Shuni virus (SHUV) is a neglected virus with zoonotic potential that was recently associated with severe disease in livestock and wildlife. Isolations of SHUV from field-collected biting midges and mosquitoes suggests that SHUV may be transmitted by these insects. Laboratory-reared biting midge species (*Culicoides nubeculosus* and *C*. *sonorensis*) and mosquito species (*Culex pipiens pipiens* and *Aedes aegypti*), that are known to transmit other arboviruses, were exposed to SHUV via an infectious blood meal. SHUV was able to successfully disseminate in both biting midge species, whereas no evidence of infection or transmission in both mosquito species was found. Our results show that SHUV infects and disseminates in two different *Culicoides* species, suggesting that these insects could play an important role in the disease transmission cycle.

## Introduction

Arthropod-borne (arbo)viruses continue to pose a threat to human and animal health [1, 2]. In particular the order *Bunyavirales* comprises emerging pathogens such as Crimean-Congo haemorrhagic fever virus (CCHFV) and Rift Valley fever virus (RVFV) [3, 4]. The World Health Organization (WHO) has included both CCHFV and RVFV to the “Blueprint” list of ten prioritized viruses likely to cause future epidemics and for which insufficient countermeasures are available [5]. In the veterinary field, prioritized viral diseases of animals, including RVFV, are notifiable to the World Organization for Animal Health (Office International des Epizooties, OIE). Apart from pathogens that are recognised as major threats by WHO and OIE, many have remained largely neglected. Before the turn of the century, West Nile virus, chikungunya virus, and Zika virus were among these neglected viruses until they reminded us how fast arboviruses can spread in immunologically naïve populations [2]. Although these outbreaks came as a surprise, in hindsight, smaller outbreaks in previously unaffected areas could have been recognised as warning signs.

Shuni virus (SHUV; family *Peribunyaviridae*, genus *Orthobunyavirus*, Simbu serogroup) recently emerged in two very distant areas of the world [6]. SHUV was isolated for the first time from a slaughtered cow in the 1960s in Nigeria [7]. During subsequent years, the virus was isolated on several occasions from domestic animals including cattle, sheep, goats, and horses [7–10], from wild animals including crocodiles and rhinoceros [10], and from field-collected *Culicoides* biting midges and mosquitoes [8, 11, 12]. More recently, SHUV was associated with malformed ruminants in Israel [13, 14]. Emergence of SHUV in areas outside Sub-Saharan Africa shows the potential of this virus to spread to new areas, and increases the risk for SHUV outbreaks in bordering territories such as Europe. Isolation of SHUV from a febrile child and detection of antibodies in 3.9% of serum samples from veterinarians in South Africa shows that SHUV can infect humans as well, although its ability to cause human disease is still uncertain [7, 15, 16].

Proper risk assessments rely on accurate knowledge of disease transmission cycles. Arbovirus transmission cycles can only become established when competent vectors and susceptible hosts encounter under suitable climatic conditions. Although SHUV has been isolated from pools of field-collected *Culicoides* biting midges and mosquitoes [7, 11, 12], the role of both insect groups as actual vectors remains to be confirmed. Detection of virus in field-collected insects is not sufficient to prove their ability to transmit the virus. Arboviruses need to overcome several barriers (*i*.*e*. midgut and salivary gland barriers) inside their vector, before they can be transmitted [17, 18]. In addition to virus isolation from field-collected vectors, laboratory studies are therefore needed to experimentally test the ability of blood-feeding insects to become infected with, maintain, and successfully transmit arboviruses (*i*.*e*., vector competence) [19]. To gain insights into the potential of *Culicoides* biting midges and mosquitoes to function as vectors of SHUV, we studied the susceptibility of four main arbovirus vector species (*Culicoides nubeculosus* and *C*. *sonorensis* biting midges, and *Culex pipiens* biotype *pipiens* and *Aedes aegypti* mosquitoes) for SHUV.

## Methods

### Cell culture

African green monkey kidney cells (Vero E6; ATCC CRL-1586) were cultured in Eagle’s minimum essential medium (Gibco, Carlsbad, CA, United States) supplemented with 5% fetal bovine serum (FBS; Gibco), 1% non-essential amino acids (Gibco), 1% L-glutamine (Gibco), and 1% antibiotic/antimycotic (Gibco). Cells were cultured as monolayers and maintained at 37°C with 5% CO_2_.

Vero E6 cells that were used in biting midge and mosquito infection experiments in the biosafety level 3 (BSL3) facility were cultured in Dulbecco's modified Eagle medium (Gibco) supplemented with 10% FBS, penicillin (100 U/ml; Sigma-Aldrich, Saint Louis, MO, United States), and streptomycin (100 μg/ml; Sigma-Aldrich). Prior to infections in the BSL3 facility, Vero E6 cells were seeded in 4-(2-hydroxyethyl)-1-piperazineethanesulfonic acid-buffered DMEM medium (HEPES-DMEM; Gibco) supplemented with 10% FBS, penicillin (100 U/ml), and streptomycin (100 μg/ml), fungizone (50 μg/ml; Invitrogen, Carlsbad, United States), and gentamycin (50 μg/ml; Gibco).

C6/36 cells (ATCC CRL-1660), derived from *Ae*. *albopictus* mosquitoes, were cultured in Leibovitz-15 (L-15) growth medium (Sigma-Aldrich) supplemented with 10% FBS, 2% Tryptose Phosphate Broth (Gibco), 1% non-essential amino acids solution, and 1% antibiotic/antimycotic. Cells were cultured as monolayers and incubated at 28°C in absence of CO_2_.

KC cells, derived from embryos of colonized *C*. *sonorensis* biting midges [20], were cultured as monolayers in modified Schneider’s *Drosophila* medium (Lonza, Basel, Switzerland) with 15% FBS, and 1% antibiotic/antimycotic at 28°C in absence of CO_2_.

### Virus

SHUV (strain An10107, P2 Vero, 1980) was kindly provided by the World Reference Center for Emerging Viruses and Arboviruses (WRCEVA). The virus was originally isolated from the blood of a slaughtered cow in 1966 in Nigeria by inoculation of neonatal mice, and passaged twice in Vero cells [21]. The passage 3 (P3) stock was generated by inoculation of Vero E6 cells with the P2 stock at a multiplicity of infection (MOI) of 0.001. The supernatant was harvested at 6 days post inoculation, centrifuged, and stored in aliquots at -80°C. The P4 stock was generated by inoculating Vero E6 cells at MOI 0.01 using the P3 stock. At this MOI, full cytopathic effect (CPE) was present at 3 days post infection. Virus titers were determined using endpoint dilution assays (EPDA) on Vero E6 cells [22]. Titers were calculated using the Spearman-Kärber algorithm and expressed as 50% tissue culture infective dose (TCID_50_) [23, 24]. The virus detection and titration procedure was validated using a SHUV-specific reverse transcriptase quantitative PCR (RT-qPCR; S1 Supporting Information).

### Growth curves

Cells were seeded in T25 cell culture flasks at densities of 7.5 × 10^5^ (Vero E6), 1.5 × 10^6^ (C6/36), or 2.5 × 10^6^ (KC cells) per flask in 10 ml complete medium. After overnight incubation, the flasks were inoculated with SHUV at an MOI of 0.01 (P4 stock). The MOI calculation for each cell line was based on the virus titer that was determined on Vero E6 cells. One hour after inoculation, the medium was removed and replaced with fresh medium. At time points 0 (sample taken directly after medium replacement), 24, 48, and 72 h post infection, 200 μl samples were taken and stored at -80°C for later analysis. For each cell line, virus titers were determined in triplicate per time point by EPDA using Vero E6 cells, which showed distinct CPE [22].

### Insect rearing

*Culicoides nubeculosus* were kindly provided by The Pirbright Institute, Pirbright laboratories, United Kingdom, in 2012 [25], and were maintained at 23°C with 16:8 light:dark cycle and 60% relative humidity. *Culicoides sonorensis* were kindly provided by the Arthropod-Borne Animal Diseases Research Laboratory, USDA-ARS (courtesy of Dr. Barbara Drolet) in 2017 [26], and were maintained at 25°C with 16:8 light:dark cycle and 70% relative humidity. Similar rearing protocols were used for both biting midge species. Eggs were transferred to square larval holding trays (*C*. *nubeculosus*: 25 x 25 x 8 cm, Kartell, Noviglio, Italy; *C*. *sonorensis*: 19 x 19 x 20 cm, Jokey, Wipperfürth, Germany) with filter wool (Europet Bernina International, Gemert-Bakel, The Netherlands) attached with double-sided tape to the bottom. Trays were filled with tap water, a few millilitres of rearing water in which larvae had completed their life cycle, and two drops of Liquifry No.1 (Interpet, Dorking, United Kingdom). Larvae were fed with a 1:1:1 mixture of bovine liver powder (MP biomedicals, Irvine, CA, US), ground rabbit food (Pets Place, Ede, The Netherlands), and ground koi food (Tetra, Melle, Germany). *Culicoides nubeculosus* larvae were additionally fed with nutrient broth No. 2 (Oxoid, Hampshire, UK). Pupae were transferred to plastic buckets (diameter: 12.2 cm, height: 12.2 cm; Jokey) and closed with netting on the top through which the biting midges could feed. Emerged adults were provided with 6% glucose solution *ad libitum*. Cow blood (Carus, Wageningen, The Netherlands) was provided through a Parafilm M membrane using the Hemotek PS5 feeding system (Discovery Workshops, Lancashire, United Kingdom) for egg production.

The *Cx*. *pipiens pipiens* colony was established in the laboratory from egg rafts collected in the field in The Netherlands during August 2016. Egg rafts were individually hatched in tubes. Pools of approximately 10 first instar larvae were identified to the biotype level using real-time PCR [27]. The colony was started by grouping larvae from 93 egg rafts identified as the *pipiens* biotype. Mosquitoes were maintained at 23°C with 16:8 light:dark cycle and 60% relative humidity [28, 29]. Adult mosquitoes were kept in Bugdorm-1 rearing cages and maintained on 6% glucose solution *ad libitum*. Cow blood or chicken blood (Kemperkip, Uden, The Netherlands) was collected in BC Vacutainer lithium heparin-coated blood collection tubes (Becton Dickinson, Breda, The Netherlands), and stored at 4°C. Blood was provided through a Parafilm M membrane using the Hemotek PS5 feeding system for egg production. Egg rafts were transferred to square larval holding trays (25 x 25 x 8 cm, Kartell) filled with tap water and two drops of Liquifry No. 1. Hatched larvae were fed with a 1:1:1 mixture of bovine liver powder, ground rabbit food, and ground koi food. Pupae were collected every 2 days and placed in Bugdorm-1 insect rearing cages.

*Aedes aegypti* mosquitoes from the Rockefeller strain (Bayer AG, Monheim, Germany) were used in all experiments. The mosquito colony was maintained as described before [30]. In short, mosquitoes were maintained at 27°C with 12:12 light:dark cycle and 70% relative humidity. Adult mosquitoes were kept in Bugdorm-1 rearing cages and maintained on 6% glucose solution *ad libitum*. Human blood (Sanquin Blood Supply Foundation, Nijmegen, The Netherlands) was provided through a Parafilm M membrane using the Hemotek PS5 feeding system for egg production. Eggs were transferred to transparent square larval holding trays (19 x 19 x 20 cm, Jokey), filled for approximately one-third with tap water and three drops of Liquifry No. 1. Hatched larvae were fed with Tetramin Baby fish food (Tetra). Larval trays were closed with fine-meshed netting, to allow adult mosquitoes to emerge inside larval trays. Twice a week, adults were aspirated from the larval trays and collected in Bugdorm-1 insect rearing cages.

### Feeding of biting midges and mosquitoes with SHUV infectious blood

Groups of adult *C*. *nubeculosus* (1–7 days old), *C*. *sonorensis* (1–11 days old), *Cx*. *p*. *pipiens* (4–20 days old), and *Ae*. *aegypti* (4–7 days old) were transferred to plastic buckets (diameter: 12.2 cm, height: 12.2 cm; Jokey) and closed with netting before being taken to the BSL3 facility. *Culex p*. *pipiens* mosquitoes were kept on water for 3 days, whereas the other species were maintained on 6% glucose solution until being offered an infectious blood meal. SHUV P3 stock with a mean titer of 3.0 x 10^6^ TCID_50_/ml was mixed 1:1 with cow blood. The used cow blood was tested negative for Schmallenberg virus (SBV) antibodies, to prevent cross-neutralisation with SHUV. The infectious blood meal was provided through a Parafilm M membrane using the Hemotek PS5 feeding system, under dark conditions at 24°C and 70% relative humidity.

After 1 h, insects were anesthetized with 100% CO_2_ and kept on a CO_2_-pad to select fully engorged females. For each species, five fully engorged females were directly stored at -80°C for each replicate. These samples were used to determine the ingested amounts of SHUV for each species. All remaining and fully engorged females were placed back into buckets with a maximum group size of 110 individuals per species per bucket. All insects were provided with 6% glucose solution via a soaked ball of cotton wool on top of the netting *ad libitum*. *Culicoides sonorensis* and *Ae*. *aegypti* were kept at 28°C for 10 days, whereas *C*. *nubeculosus* and *Cx*. *p*. *pipiens* were kept at 25°C for 10 days. These temperatures were selected for optimal replication of the virus, and to reflect differences in the rearing temperature for each species. Three replicate experiments of *C*. *nubeculosus* (N_1_ = 84, N_2_ = 82, N_3_ = 77, N_total_ = 243), *C*. *sonorensis* (N_1_ = 9, N_2_ = 9, N_3_ = 30, N_total_ = 48), and *Cx*. *p*. *pipiens* (N_1_ = 89, N_2_ = 57, N_3_ = 65, N_total_ = 211) were carried out, and two replicate experiments of *Ae*. *aegypti* (N_1_ = 72, N_2_ = 77, N_total_ = 149). During each replicate, biting midges and mosquitoes were fed in parallel with the same infectious blood meal.

### Intrathoracic injections of mosquitoes with SHUV

Adult female *Cx*. *p*. *pipiens* (3–9 days old) and *Ae*. *aegypti* (4–6 days old) mosquitoes were injected with SHUV into the thorax to investigate the role of mosquito barriers on dissemination of SHUV. Mosquitoes were anesthetized with 100% CO_2_ and positioned on the CO_2_-pad. Female mosquitoes were intrathoracically injected with 69 nl of SHUV (P3 stock with a titer of 3.0 x 10^6^ TCID_50_/ml) using a Drummond Nanoject II Auto-Nanoliter injector (Drummond Scientific, Broomall, Unites States). Injected *Cx*. *p*. *pipiens* were maintained at 25°C and injected *Ae*. *aegypti* were maintained at 28°C. Mosquitoes were incubated for 10 days at the respective temperatures, and had access to 6% glucose solution *ad libitum*. Injections were done during a single replicate experiment for *Cx*. *p*. *pipiens* (N = 50) and *Ae*. *aegypti* (N = 50).

### Infectivity assays

After 10 days of incubation at the respective incubation temperatures, samples from surviving biting midges and mosquitoes were collected. Biting midges were anesthetized with 100% CO_2_ and transferred individually to 1.5 ml Safe-Seal micro tubes (Sarstedt, Nümbrecht, Germany) containing 0.5 mm zirconium beads (Next Advance, Averill Park, NY, United States). For a selection of *C*. *nubeculosus* (N = 77) and *C*. *sonorensis* (N = 30) from one replicate experiment, heads were removed from bodies and separately stored in tubes. All samples were stored at -80°C until further processing.

Mosquitoes were anesthetized with 100% CO_2_ to remove legs and wings. Mosquito saliva was then collected by inserting the proboscis into a 200 μl yellow pipette tip (Greiner Bio-One) containing 5 μl of a 1:1 solution of 50% glucose solution and FBS. The saliva sample was transferred to a 1.5 ml micro tube containing 55 μl of fully supplemented HEPES-DMEM medium. Mosquito bodies were individually stored in 1.5 ml Safe-Seal micro tubes containing 0.5 mm zirconium beads.

Frozen biting midge and mosquito tissues were homogenized for 2 min at maximum speed (setting 10) in the Bullet Blender Storm (Next advance), centrifuged for 30 seconds at 14,500 rpm in the Eppendorf minispin plus (Eppendorf, Hamburg, Germany), and suspended in 100 μl of fully supplemented HEPES-DMEM medium. After addition of the medium, samples were blended again for 2 min at maximum speed, and centrifuged for 2 min at 14,500 rpm. Mosquito saliva samples were thawed at RT and vortexed before further use. In total 30 μl of each body or saliva sample was inoculated on a monolayer of Vero E6 cells in a 96 wells plate. SHUV stock or infectious blood mixture was included as positive control and wells to which no sample was added were included as negative controls. After 2–3 h the inoculum was removed and replaced by 100 μl of fully supplemented HEPES-DMEM medium. Wells were scored for virus induced CPE at 3 and 7 days post inoculation, with full CPE being observed at the latter time point. Afterwards, virus titers for positive samples of biting midge bodies and heads, as well as mosquito bodies and saliva were determined with single EPDA on Vero E6 cells [30]. Virus titers were determined using the Reed & Muench algorithm [31]. A subset of samples was validated by RT-qPCR, to confirm that observed CPE was induced by SHUV (S1 Supporting Information).

Infection rate (virus-infected whole body) and dissemination efficiency (virus-infected head) were determined for biting midges, whereas infection rate (virus-infected whole body) and transmission efficiency (virus-infected saliva) were determined for mosquitoes. Infection rate, dissemination efficiency, and transmission efficiency were calculated, respectively, by dividing the number of females with virus-infected bodies (infection), virus-infected heads (dissemination), or virus-infected saliva (transmission) by the total number of females tested in the respective treatment and that survived the incubation period. The values were subsequently expressed as percentages by multiplying with 100. Two biting midge samples of which only the head was virus-positive, but not the body, were considered to be uninfected.

## Results

### Efficient growth of SHUV in mammalian, mosquito, and midge cells

**Fig 1:**
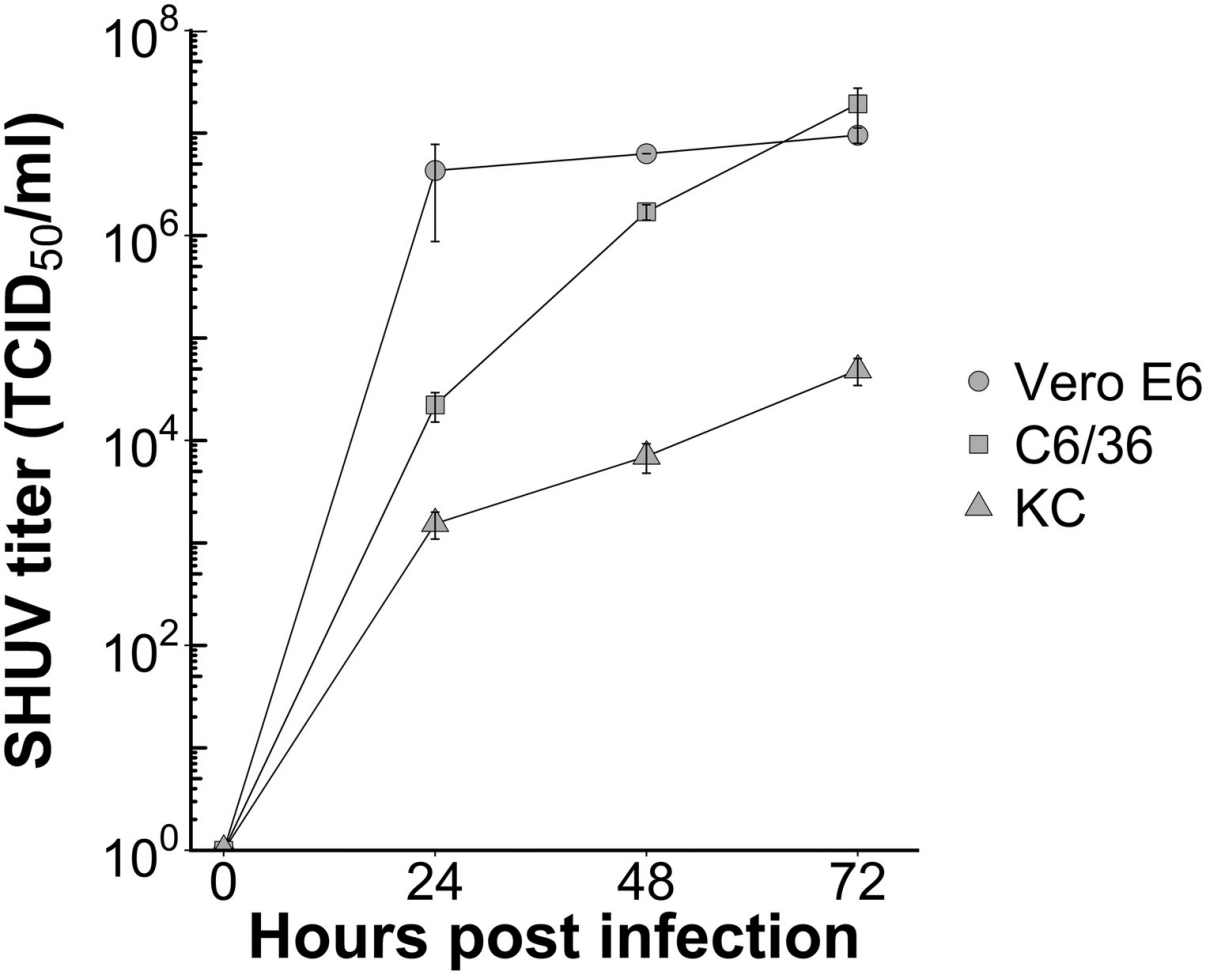
Growth of Shuni virus (SHUV) in mammalian (Vero E6), mosquito (C6/36), and *Culicoides* biting midge (KC) cells. Three different cell lines (African green monkey (Vero E6) cells, *Aedes albopictus* (C6/36) cells, and *Culicoides sonorensis* (KC) cells) were inoculated with SHUV at an MOI of 0.01, and kept at 28°C (C6/36 and KC) or 37°C (Vero E6). Virus titers were determined at time points 0, 24, 48, and 72 h post infection. Mean virus titers ± SEM for three replicates are shown.

Mammalian, mosquito, and midge cells were inoculated with SHUV to gain insight into the replicative fitness of this virus and strain in different host cell types. The results show that SHUV is capable to produce progeny in all three cell types (Fig 1 and S1 Data). Of note, a strong CPE was observed in the Vero E6 cells upon infection whereas no CPE was observed in the insect cell lines. Therefore, Vero E6 cells were used to determine titers by EPDA.

### *Culicoides* biting midges are highly susceptible to SHUV infection

To evaluate the susceptibility of two species of biting midges (*C*. *nubeculosus* and *C*. *sonorensis*) for SHUV, groups of individuals of both species were orally exposed to an infectious blood meal with a mean SHUV titer of 3.0 x 10^6^ TCID_50_/ml. SHUV titers of ingested blood were determined for a selection of 10 fully engorged females for each species, that were directly stored at -80°C after feeding. Both species ingested low amounts of SHUV that were below the detection limit of the endpoint dilution assay of 10^3^ TCID_50_/ml.

**Fig 2:**
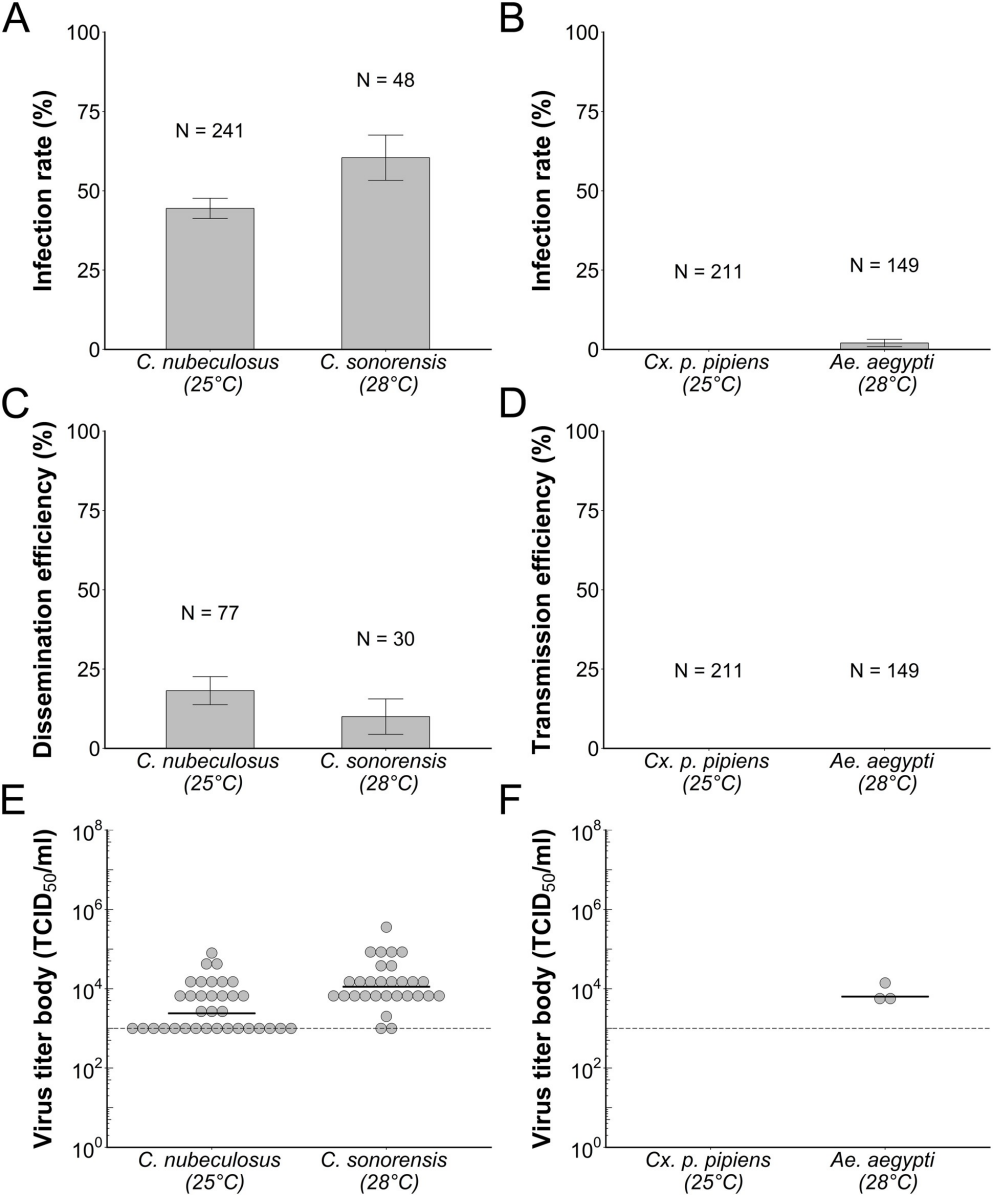
Susceptibility of orally exposed biting midges and mosquitoes for Shuni virus (SHUV). Mean infection rates of *Culicoides nubeculosus* (A; N = 243, 25°C), *C*. *sonorensis* (A; N = 48, 28°C), *Culex pipiens pipiens* (B; N = 211, 25°C), and *Aedes aegypti* (B; N = 149, 28°C) orally exposed to SHUV after 10 days of incubation at the respective temperatures. Infection rate represents the percentage of virus-positive females out of the total number of blood-fed females that remained alive at the end of the incubation period. Error bars indicate the SEM. Mean dissemination efficiency (C) of *C*. *nubeculosus* (N = 77, 25°C) and *C*. *sonorensis* (N = 30, 28°C). Dissemination efficiency represents the percentage of biting midge females with virus-positive heads out of the total number of blood-fed female biting midges that were alive at the end of the incubation period. Mean transmission efficiency (D) of *Cx*. *p*. *pipiens* (N = 211, 25°C) and *Ae*. *aegypti* mosquitoes (N = 149, 28°C). Transmission efficiency represents the percentage of female mosquitoes with virus-positive saliva out of the total number of blood-fed female mosquitoes that were alive at the end of the incubation period. Error bars indicate the SEM. SHUV titers of virus-positive bodies of *C*. *nubeculosus* (E; N = 34, 25°C), *C*. *sonorensis* (E; N = 29, 28°C), and *Ae*. *aegypti* (F; N = 3, 28°C) after 10 days incubation at the respective temperatures. Each dot represents one individual female, and the black bar indicates the median. The detection limit of the endpoint dilution assay is indicated with the dashed line.

Infection rates were also determined after 10 days of incubation at temperatures of 25°C (*C*. *nubeculosus* and *Cx*. *p*. *pipiens*) or 28°C (*C*. *sonorensis* and *Ae*. *aegypti*; Fig 2 and S2 Data). Both biting midge species showed high infection rates of 44% for *C*. *nubeculosus* (N = 243), and 60% for *C*. *sonorensis* (N = 48; Fig 2A). SHUV replicated to median titers of 2.4 x 10^3^ TCID_50_/ml in body samples of *C*. *nubeculosus* and 1.1 x 10^4^ TCID_50_/ml in body samples of *C*. *sonorensis* (Fig 2E). For one replicate experiment, heads were separated from the bodies and tested for presence of SHUV to assess whether the virus successfully passed from the midgut to the haemocoel, indicative of dissemination throughout the body. Dissemination efficiencies were 18% (N = 77) for *C*. *nubeculosus* and 10% (N = 30) for *C*. *sonorensis* (Fig 2C). In all virus-positive heads that induced CPE, SHUV titers were lower than 10^3^ TCID_50_/ml. Because only very low amounts of SHUV were detected in biting midge heads, the actual percentage of disseminated infections might be higher. A subset of the samples was additionally tested by RT-qPCR to confirm that CPE was induced by SHUV (S1 Supporting Information). The relatively high infection rates and dissemination efficiencies observed in this study and the absence of a salivary glands barrier in biting midges as shown in previous studies [17, 32], suggests that both *C*. *nubeculosus* and *C*. *sonorensis* have the potential to transmit SHUV.

### Low susceptibility of mosquitoes to SHUV

SHUV was previously isolated from field-collected mosquitoes [8]. Therefore, we determined vector competence for two mosquito species (*Cx*. *p*. *pipiens* and *Ae*. *aegypti*) which are important vectors for several arboviruses [22, 28, 30]. SHUV titers of ingested blood were determined for a selection of 10 fully engorged female mosquitoes that were directly stored at -80°C after feeding on an infectious blood meal with a SHUV titer of 3.0 x 10^6^ TCID_50_/ml. Similar to results obtained with the biting midges, the amounts of SHUV ingested by both mosquito species was less than 10^3^ TCID_50_/ml.

No SHUV infection was observed in the *Cx*. *p*. *pipiens* mosquitoes (N = 211) following oral exposure, whereas infection rates of 2% were found for orally exposed *Ae*. *aegypti* mosquitoes (N = 149; Fig 2B). SHUV replicated to median titers of 6.3 x 10^3^ TCID_50_/ml in body samples of *Ae*. *aegypti* (Fig 2F), which was comparable to titers found in biting midges. No SHUV was detected in any of the saliva samples taken from either *Cx*. *p*. *pipiens* or *Ae*. *aegypti* (Fig 2D). Thus, SHUV was able to successfully infect a small proportion of *Ae*. *aegypti* mosquitoes but not *Cx*. *p*. *pipiens*, and no evidence was found for transmission of SHUV by mosquitoes.

**Fig 3:**
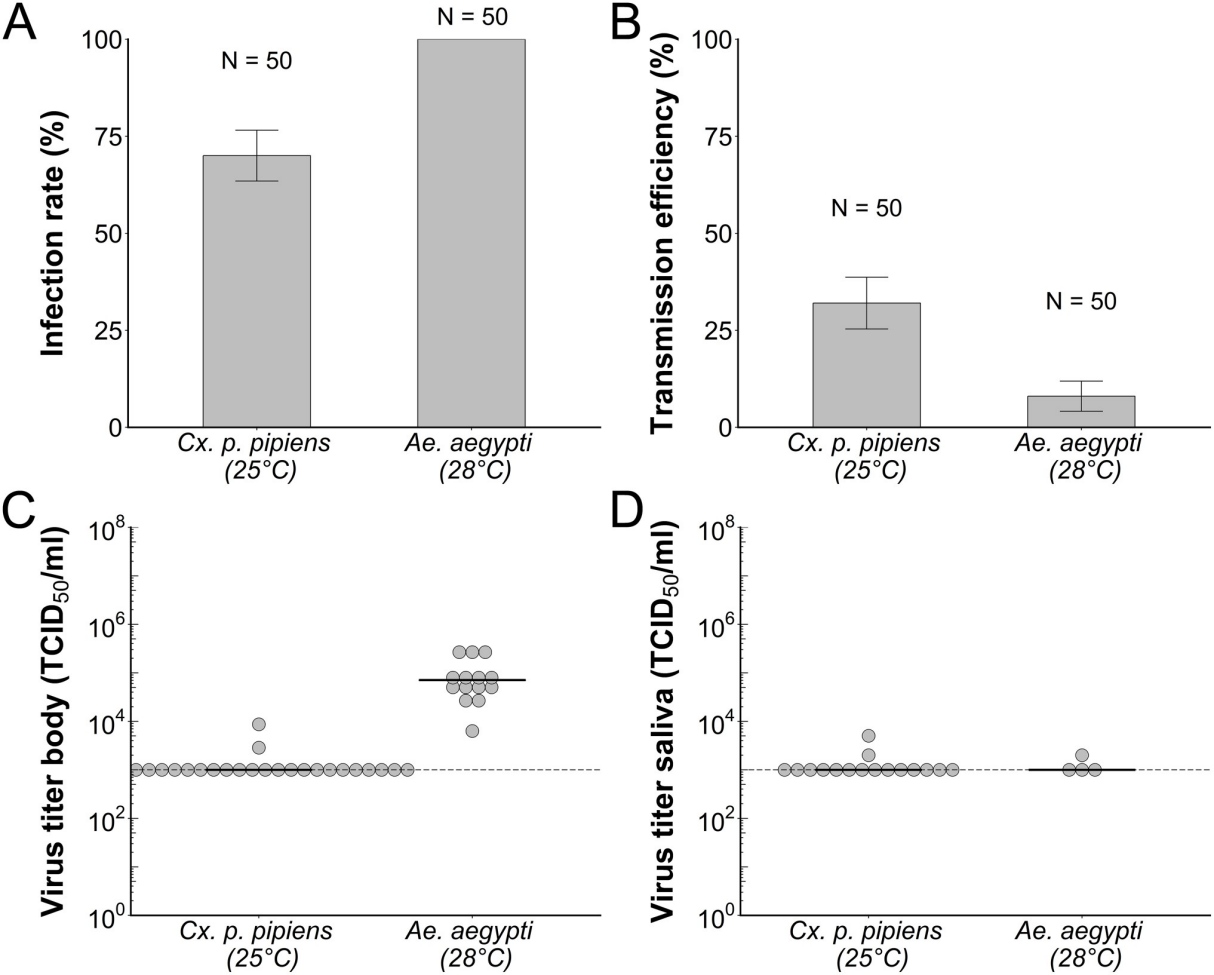
Susceptibility of intrathoracically injected mosquitoes for Shuni virus (SHUV). Mean infection rates (A) and transmission efficiencies (B) of *Culex pipiens pipiens* (N = 50) and *Aedes aegypti* (N = 50) intrathoracically injected with SHUV after 10 days incubation at 25°C and 28°C, respectively. Infection rate represents the percentage of females with virus-positive bodies out of the total number of injected female mosquitoes that were alive at the end of the incubation period. Transmission efficiency represents the percentage of females with virus-positive saliva out of the total number of injected female mosquitoes that were alive at the end of the incubation period. Error bars indicate the SEM as calculated for technical replicates. SHUV titers of virus-positive body samples (C) and saliva samples (D) of *Cx*. *p*. *pipiens* (body samples: N = 26, saliva samples: N = 16) and *Ae*. *aegypti* (body samples: N = 14, saliva samples: N = 4) intrathoracically injected with SHUV after 10 days incubation at 25°C and 28°C, respectively. Each dot represents an individual sample, and the black bar indicates the median. The detection limit of the endpoint dilution assay is indicated with the dashed line.

The very low infection rates of mosquitoes triggered further investigation into potential mosquito barriers against SHUV infection. To this end, *Cx*. *p*. *pipiens* and *Ae*. *aegypti* mosquitoes were intrathoracically injected with SHUV, to bypass the potential midgut barrier. Direct injection of SHUV into the thorax resulted in high infection rates of 70% for *Cx*. *p*. *pipiens* (N = 50), and 100% for *Ae*. *aegypti* (N = 50; Fig 3A). Transmission efficiency of 32% (N = 50) was found for *Cx*. *p*. *pipiens* and 8% (N = 50) for *Ae*. *aegypti* (Fig 3B). Interestingly, although infection rates of *Cx*. *p*. *pipiens* were below 100%, we found a relatively high transmission efficiency. This may indicate a relatively weaker salivary gland barrier in *Cx*. *p*. *pipiens* compared to *Ae*. *aegypti* mosquitoes that had 100% infection rate, but relatively low transmission efficiency.

To gain more insight in replication of SHUV, virus titers were determined for virus-infected mosquito body and saliva samples. Titers of virus-infected *Cx*. *p*. *pipiens* body samples were almost all below the detection limit of 10^3^ TCID_50_/ml of the endpoint dilution assay (Fig 3C). This indicates that even when SHUV is injected into the thorax, there is no productive virus replication. In contrast, we found median titers of 7.1 x 10^4^ TCID_50_/ml for virus-infected *Ae*. *aegypti* body samples. This shows that SHUV is able to successfully replicate in *Ae*. *aegypti* when the midgut barrier is bypassed. In the majority of mosquito saliva samples, SHUV titers were less than 10^3^ TCID_50_/ml (Fig 3D). Taken together, SHUV is able to disseminate in mosquitoes, but both the midgut and salivary glands form a barrier for SHUV.

## Discussion

SHUV was previously isolated from field-collected pools of *Culicoides* biting midges and from mosquitoes, but their involvement in SHUV transmission remained to be confirmed [8, 11, 12]. Here, we show for the first time that SHUV is able to infect and replicate in biting midges as well as in mosquitoes, but only the biting midge species evaluated in the present study can be considered highly susceptible to infection.

Both *C*. *nubeculosus* and *C*. *sonorensis* showed high infection rates of 44% and 60% when incubated for 10 days at 25°C and 28°C, respectively. It has been demonstrated that a salivary gland barrier is absent for Orbiviruses and Schmallenberg virus in biting midges [17, 32]. This knowledge, in combination with evidence of successful dissemination of SHUV to the heads indicates that the biting midge species evaluated in the present study are likely competent vectors of SHUV. Importantly, the finding that SHUV replicates efficiently in two biting midge species from a different geographic background suggests that various species of *Culicoides* may function as vectors of SHUV.

SHUV infection and replication in biting midges seems more efficient compared to other biting midge-borne viruses such as SBV and bluetongue virus (BTV), which generally show infection rates up to 30% [32–36]. Both SBV and BTV have caused sudden and large-scale epizootics in Europe, with devastating consequences for the livestock sector [37, 38]. The relatively high susceptibility and efficiency of replication in biting midges, and recent spread of SHUV to areas outside Sub-Saharan Africa [13], should therefore be interpreted as a warning for its epizootic potential.

In contrast to the high infection rates in biting midges, only few orally exposed *Ae*. *aegypti* mosquitoes became infected with SHUV during 10 days of incubation at 28°C. In addition, no evidence of successful dissemination to the salivary glands of the two mosquito species was found. SHUV replication and transmission (8%) was observed when the virus was directly injected into the thorax of *Ae*. *aegypti* mosquitoes. This indicates that both the midgut infection barrier and the salivary gland barrier prevent infection and subsequent transmission of SHUV by *Ae*. *aegypti* mosquitoes. Of the *Cx*. *p*. *pipiens* mosquitoes that were orally exposed to SHUV, none became infected during 10 days of incubation at 25°C. Moreover, replication of SHUV was low in *Cx*. *p*. *pipiens*, as evidenced by low titers when it was directly injected into the thorax. However, a relatively high percentage of mosquito saliva samples contained SHUV. We therefore conclude that the midgut barrier is the main barrier that prevents infection of *Cx*. *p*. *pipiens* with SHUV. Our findings are in line with an earlier study on the closely-related SBV, which showed no evidence for involvement of *Cx*. *pipiens* in virus transmission, although SBV was able to infect *Cx*. *pipiens* mosquitoes [39]. However, as *Cx*. *theileri* has been identified as a vector of several other bunyaviruses, this mosquito may also be a possible vector of SHUV [40, 41]. Thus, vector competence studies with additional mosquito species collected from the field are needed to fully understand the possible role of mosquitoes in natural transmission cycles of SHUV.

In this study, we determined infection, dissemination, and transmission of SHUV by infectivity assays and virus titers by EPDA (*i*.*e*. assays based on inoculation of samples on Vero cells which are then screened for CPE). Such infectivity assays and EPDAs have the advantage of detecting infectious virus particles, whereas other methods like qPCR that quantify genome equivalents, may include defective virus particles and thereby not accurately represent infectious virus. Of note, observed CPE in the infectivity assays and EPDAs was found to invariably correspond with SHUV RNA as determined by RT-qPCR (S1 Supporting Information).

Recent outbreaks of SBV and BTV exemplified the tremendous impact of midge-borne viruses on animal health [37, 38]. Our study demonstrates highly efficient infection, replication, and dissemination of SHUV in two biting midge species (*C*. *nubeculosus* and *C*. *sonorensis*). However, conclusive evidence for SHUV transmission by biting midges should be provided by experiments with infected biting midges and susceptible mammals, although these kind of experiments are costly and complex. We cannot exclude that results obtained with laboratory-reared vectors are different from those obtained with field-collected vectors. Therefore, future studies should test vector competence of field-collected *Culicoides* biting midge and mosquito species exposed to different quantities of SHUV, to more accurately predict the risk of SHUV transmission in specific areas. These experiments in combination with behavioural and ecological research will contribute to our understanding of the transmission cycle of SHUV.

## Supporting information

S1 Supporting Information RT-qPCR validation of Shuni virus read-out based on cytopathic effects. (PDF)

S1 Data Dataset Shuni virus growth curves. (XLSX)

S2 Data Dataset Shuni virus vector competence experiments. (XLSX)
