# Supplementary material for: Vector competence of biting midges and mosquitoes for Shuni virus
Source: PLoS Negl Trop Dis. 2018 Dec 7;12(12):e0006993. doi: 10.1371/journal.pntd.0006993 (PMC6285265; doi:10.1371/journal.pntd.0006993)
Supplement: S1 Supporting Information — (PDF) [file pntd.0006993.s001.pdf]

Supplement 1: Validation of Shuni virus read-out based on cytopathic effects

Methods

To confirm that the observed cytopathic effects (CPE) in the infectivity and endpoint dilution assays (EPDA) was induced by Shuni virus (SHUV) a reverse transcriptase quantitative PCR (RT-qPCR) was performed on supernatants of inoculated Vero cell monolayers. A selected subset of samples including Schmallenberg virus (SBV) and SHUV stocks (positive virus controls), cell-culture medium (negative controls), non-virus exposed biting midges and mosquitoes, and SHUV-exposed biting midges and mosquitoes that tested negative or positive for SHUV infection in the infectivity assays were inoculated with a monolayer of Vero cells in a 96-well plate (Fig S1A). After 2-3 h the inoculum was removed and replaced by 100 µl of fully supplemented HEPES-DMEM medium. Wells were subsequently scored for virus-induced CPE at 7 days post inoculation. Each well was imaged through a Dino-Eye ocular (Dino-Lite Europe, Naarden, The Netherlands) using a light microscope (Fig S1B). The 100 µl supernatant was subsequently removed from each well and added to an Eppendorf tube containing 300 µl Trizol-LS (Thermo Fisher Scientific, Massachusetts, United States) to inactivate the virus. The 96-well plate was fixed with 4% paraformaldehyde and washed three times with phosphate buffered saline before the remaining cells were stained with 50 µl crystal violet (Sigma-Aldrich, Missouri, United States; Fig S1C). After 10 minutes the plate was washed three times with water and air dried before imaging. The supernatant samples in Trizol-LS were used for SHUV-specific RT-qPCR (Fig S1D).

Viral RNA was isolated from the Trizol-LS samples with the Direct-Zol™ RNA Miniprep kit (Zymo Research, California, United States) according to the manufacturer's instructions. The SHUV specific RT-qPCR was designed (PrimerQuest Tool; Integrated DNA Technologies, Iowa, United States) using the S-segment sequence (Genbank: KU937313.1). Primers and probe sequences are given in table S1. The LightCycler RNA Amplification Kit HybProbe (Roche, Almere, The Netherlands) in combination with a LightCycler 480 system (Roche) were used for a one-step RT-qPCR reaction. Cycling conditions were as follows:

reverse transcriptase at 45°C for 30 min, denaturation at 95°C for 5 min, 40 cycles of 5 s at 95°C and 35 s at 57°C.

Table S1: Shuni virus primers and probe for qPCR.

	Sequence
Forward primer	5′-GAAGGCCAAGATGGTACT-3′
Probe	5’-FAM-AGTAAGACGGCACAACCGAGTGTT-BHQ1-3’
Reverse primer	5’-CAATACACAGCAAATCCTGT-3’

Results and conclusion

As shown in Figure S1, a strong correlation between CPE and a RT-qPCR positive result was observed. All CPE presenting wells initially inoculated with a serially diluted SHUV stock presented a high signal in the PCR. Of note no specific signal was observed with a SBV serially diluted stock. Importantly, no CPE or PCR positive signal was observed with SHUV negative bodies. A high SHUV-specific PCR signal was observed with samples showing CPE. Altogether, this experiment confirms that the infectivity assays are a reliable tool for detecting infectious SHUV in biting midge and mosquito samples.

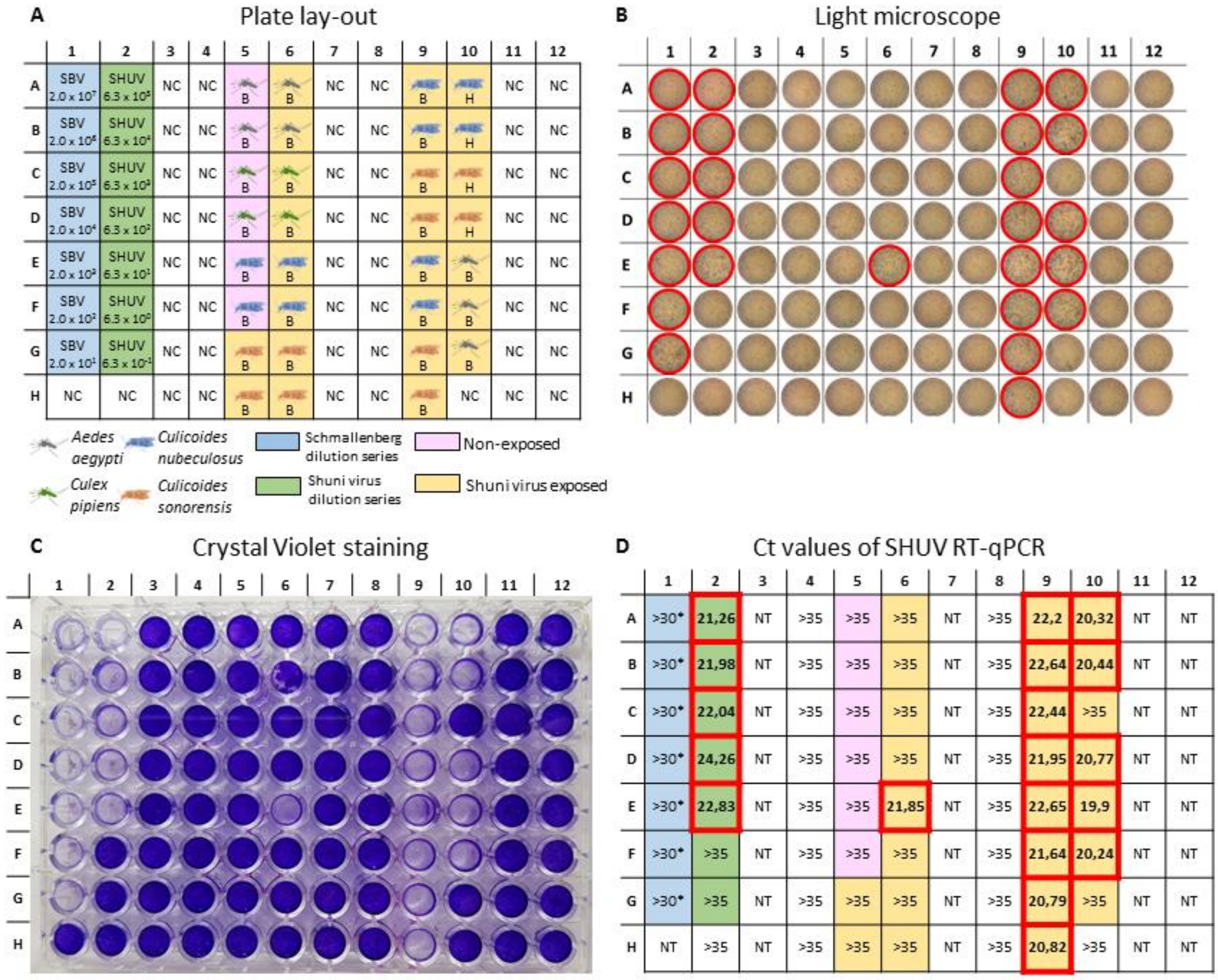

Figure S1. Validation of Shuni virus (SHUV) infection assay. Serial diluted Schmallenberg virus (SBV) and SHUV stocks, homogenates of non-exposed *Aedes aegypti, Culex pipiens* and *Culicoides nubeculosus*, and SHUV exposed vector species (*Ae. aegypti, Cx. pipiens, C. nubeculosus* and *C. sonorensis*) were added to a 96 wells plate with Vero cells as indicated in **panel A**. B = whole body, H = head, NC = negative control. **Panel B:** Light microscope images of cells at 7 days post inoculation. Red lining indicates presence of cytopathic effects. **Panel C:** Crystal violet staining of Vero cells in the 96 wells plate following the 7 days incubation. **Panel D:** SHUV RT-qPCR of the supernatants obtained at 7 days post inoculation. Red lining indicates samples with low Ct values. NT = not tested, * = very low signal with no sigmoidal amplification which is probably due to some very low level cross-reactivity between the SHUV primers and probe with the SBV S-genome segment.
